# Remodeling the hepatic fibrotic microenvironment with emerging nanotherapeutics: a comprehensive review

**DOI:** 10.1186/s12951-023-01876-5

**Published:** 2023-04-07

**Authors:** Xingtao Zhao, Felix Kwame Amevor, Xinyan Xue, Cheng Wang, Zhifu Cui, Shu Dai, Cheng Peng, Yunxia Li

**Affiliations:** 1 State Key Laboratory of Southwestern Chinese Medicine Resources, Key Laboratory of Standardization for Chinese Herbal Medicine, Ministry of Education, Chengdu university of Traditional Chinese Medicine, Chengdu, China; 2 School of Pharmacy, Chengdu University of Traditional Chinese Medicine, Chengdu, 611137 China; 3 Farm Animal Genetic Resources Exploration and Innovation Key Laboratory of Sichuan Province, Sichuan Agricultural University, Chengdu, 611130 China; 4 College of Animal Science and Technology, Southwest University, Chongqing, 400715 China; 5 No. 1166, Liu Tai Avenue, Wenjiang district, Chengdu, Sichuan China

**Keywords:** Hepatic microenvironment, Engineered nanotherapeutics, Liver fibrosis, Metabolic reprogramming, Vascular remodeling, Immunotherapy

## Abstract

Liver fibrosis could be the last hope for treating liver cancer and remodeling of the hepatic microenvironment has emerged as a strategy to promote the ablation of liver fibrosis. In recent years, especially with the rapid development of nanomedicine, hepatic microenvironment therapy has been widely researched in studies concerning liver cancer and fibrosis. In this comprehensive review, we summarized recent advances in nano therapy-based remodeling of the hepatic microenvironment. Firstly, we discussed novel strategies for regulatory immune suppression caused by capillarization of liver sinusoidal endothelial cells (LSECs) and macrophage polarization. Furthermore, metabolic reprogramming and extracellular matrix (ECM) deposition are caused by the activation of hepatic stellate cells (HSCs). In addition, recent advances in ROS, hypoxia, and impaired vascular remodeling in the hepatic fibrotic microenvironment due to ECM deposition have also been summarized. Finally, emerging nanotherapeutic approaches based on correlated signals were discussed in this review. We have proposed novel strategies such as engineered nanotherapeutics targeting antigen-presenting cells (APCs) or direct targeting T cells in liver fibrotic immunotherapy to be used in preventing liver fibrosis. In summary, this comprehensive review illustrated the opportunities in drug targeting and nanomedicine, and the current challenges to be addressed.

**Graphical Abstract:**

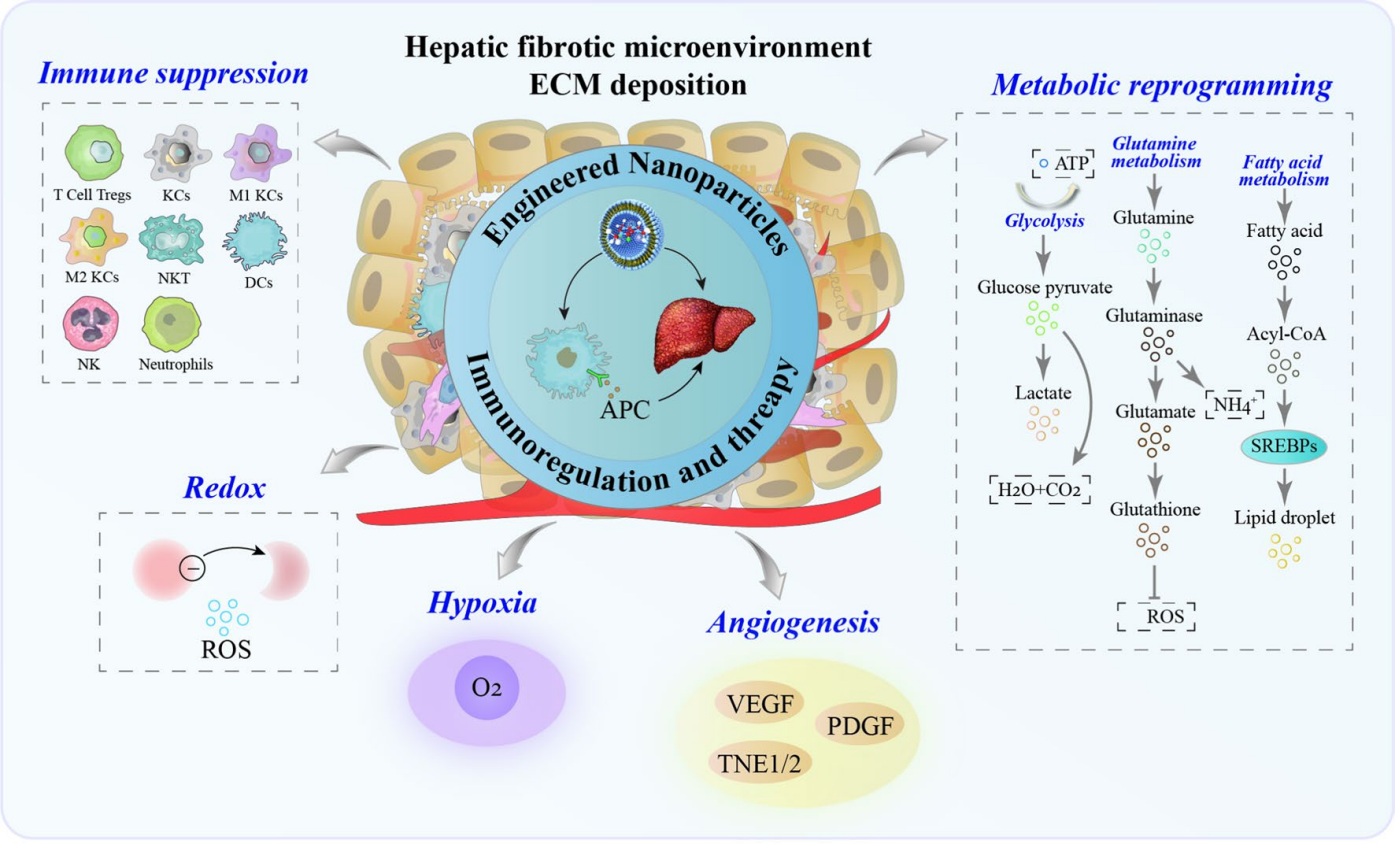

## Introduction

Chronic liver disease (CLD) is a continuous and progressive pathological condition that progresses from initial fat accumulation to hepatitis and liver fibrosis, with end-stage fibrosis known as cirrhosis, which is highly susceptible to hepatocellular carcinoma (HCC) [1]. Approximately 12% of the global population (800 million people) suffer from liver disease and causing 2 million deaths each year [2]. Cancer is a worldwide threat to public health and HCC is the second leading cause of cancer death [3]. Is cancer curable? Almost all CLDs have provided convincing evidence confirming the reversibility of liver fibrosis and in clinical trials, in patients with chronic viral hepatitis and patients with advanced disease, timely "braking" interventions can subside fibrosis, which may be the last straw in the treatment of cancer [4–6].

**Fig. 1:**
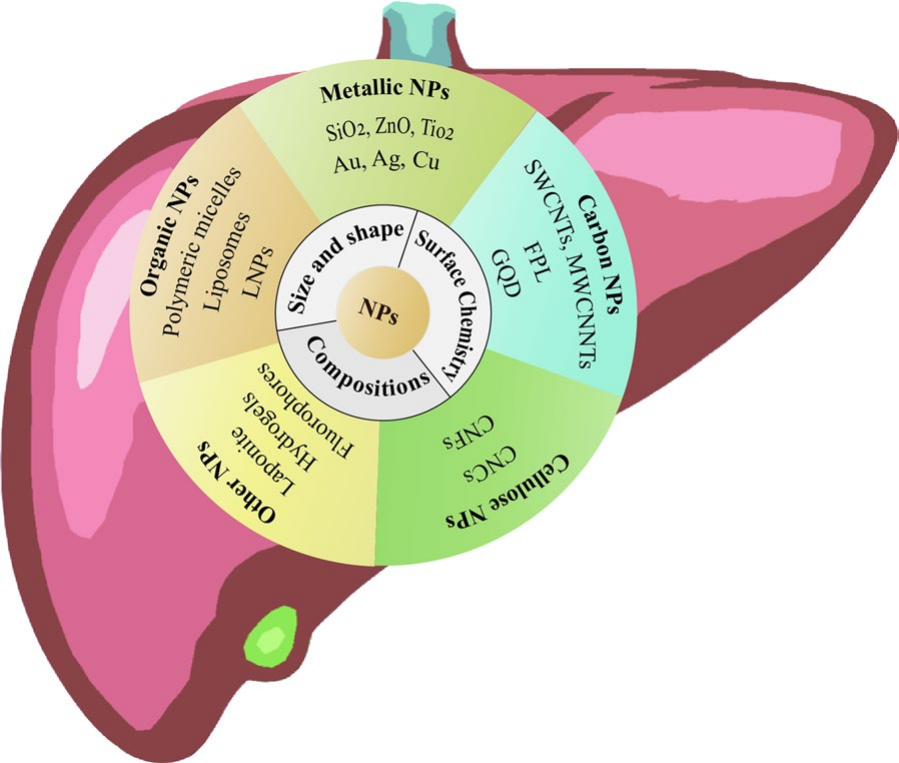
The major intrinsic properties of NPs and cellular uptake by the major liver cells

The liver is highly complex and consists of two different cell entities, parenchymal cells, and non-parenchymal cells. Parenchymal cells include hepatocytes (60–70%) and cholangiocytes and account for most of the metabolic liver functions. While the non-parenchymal cells constitute 30–40% of total liver cells, like LSECs, HSCs, KCs, and other immune cells, playing a central role in the physiological processes of the liver [7]. The liver microenvironment, which fosters the survival and activity of liver cells, plays an important role in maintaining the normal structure and physiological function of the liver. The homeostasis of the liver microenvironment is disrupted during liver fibrosis development, causing hepatocyte damage, LSECs capillarization, HSCs activation, macrophage polarization, and immune cell suppression, and changing the cell–cell and cell–matrix interactions, which eventually form the hepatic fibrotic microenvironment [7, 8]. Recent studies have shown that modest prognostic performance (area under the receiver operating characteristic curve from 0.54 to 0.71) of five indirect markers of fibrosis (aspartate aminotransferase [AST]–to-platelet ratio index [APRI], Fibrosis-4 Index [FIB-4], BARD, Forns, NAFLD score [NAS]) [9] and direct markers-Liver Fibrosis test (LF) [10] to predict future development of cirrhosis and severe liver disease in the general population. And liver fibrosis can be assessed with relatively high accuracy noninvasively by serological tests, transient elastography, and radiological methods. These modalities may be utilized for screening for liver fibrosis in at-risk populations [11]. Nanoparticle (NPs) delivery systems have been widely studied as a drug delivery strategy in drug research and the liver targeting of NPs may be some of the nanomaterials beneficial for liver disease therapy [12], as shown in Fig. 1. NPs have diverse compositions, such as metallic NPs including metal (Ag and Au nanoparticles) and metal oxide (MOx) NPs including transition-metal oxides (TMOs, e.g., SiO2, ZnO, and TiO2), carbon NPs including 1D carbon nanotubes (CNTs) and 2D graphene-based NPs (FPL, and GO), cellulose nanocrystal (CNC) and cellulose nanofiber (CNF), Fluorescent NPs (quantum dots, CDs, Organic fluorophores), and organic NPs including lipid NPs, liposomes, and polymer NPs.

**Table 1:** Preclinical NPs for the treatment of liver fibrosis

NP type	Target cell	Effect	Refer.
Anionic HA-containing NPs	LSECs, Tregs	Suppressing antigen-specific immune responses	[29]
AB diblock copolymers NPs	LSECs, KCs	Leading to immune inflammation	[33]
Stab2-containing NPs	LSECs, Tregs	Clearing of lipoproteins	[30]
Mannose-modified albumin NPs	Macrophages	TGFβ-siRNA to CD206 + as an anti-fibrotic strategy	[37]
CMC-containing NPs	Macrophages	Reducing host immune response	[40]
PS-containing NPs	Macrophages	Reducing collagen fiber deposition	[42]
silk or silicon-containing NPs	Macrophages	Pro-inflammatory phenotype	[69]
hydrogel particles	M2-polarized macrophages	Improving immunocompromised and impaired angiogenesis	[108, 109]
DEX/HA-TK-ART PMs	M2-polarized macrophages	HIF-1α/NF-κB signaling cascade	[90]
mLNP-siHMGB1	Macrophages, HSCs	Inhibiting the activation of HSCs	[38]
DSPE-PEG-CeO2 NPs	Macrophages, HSCs	Reducing inflammation	[86]
CNF	HSCs, macrophages	Increasing glycolysis and reprogramming and reducing initiated inflammatory	[70]
VA-SLNs	HSCs	Reducing PPARγ/SREBPs-mediated lipid accumulation	[61]
CS sulfate PMs	HSCs	Anti-fibrotic strategy	[63]
CS-coated green silver NPs	HSCs	Bounding to the fibrogenic protein TGF-β	[65]
hydrogels	HSCs	Blocking the TGF-β1/Smad pathway	[66]
RGD	HSCs	Inhibiting the proliferation of HSCs	[82]
Chol-PCX/miRNA NPs	HSCs, T cells	Disrupting the lipid metabolic network	[73, 74]
GQD	HSCs	Inhibiting lipid peroxidation, apoptosis, and autophagy	[87]
CeO2 NPs	HSCs	Antioxidant effect	[85]
FPL	HSCs	Antioxidant effect	[88]
ChiBil carrying losartan	HSCs	attenuating iron death	[89]
PEG-PLGA-containing NPs	LSECs, hepatocytes and HSCs	Constricting abnormal blood vessels, reducing MVD	[99]
Self-assembled PMs	LSECs, hepatocytes and HSCs	Avoiding the hepatotoxicity and side effects	[96]
NPs laponite	HUVECs	enhancing HIF-1α and VEGF expression, accelerating neovascularization	[110]

**Fig. 2:**
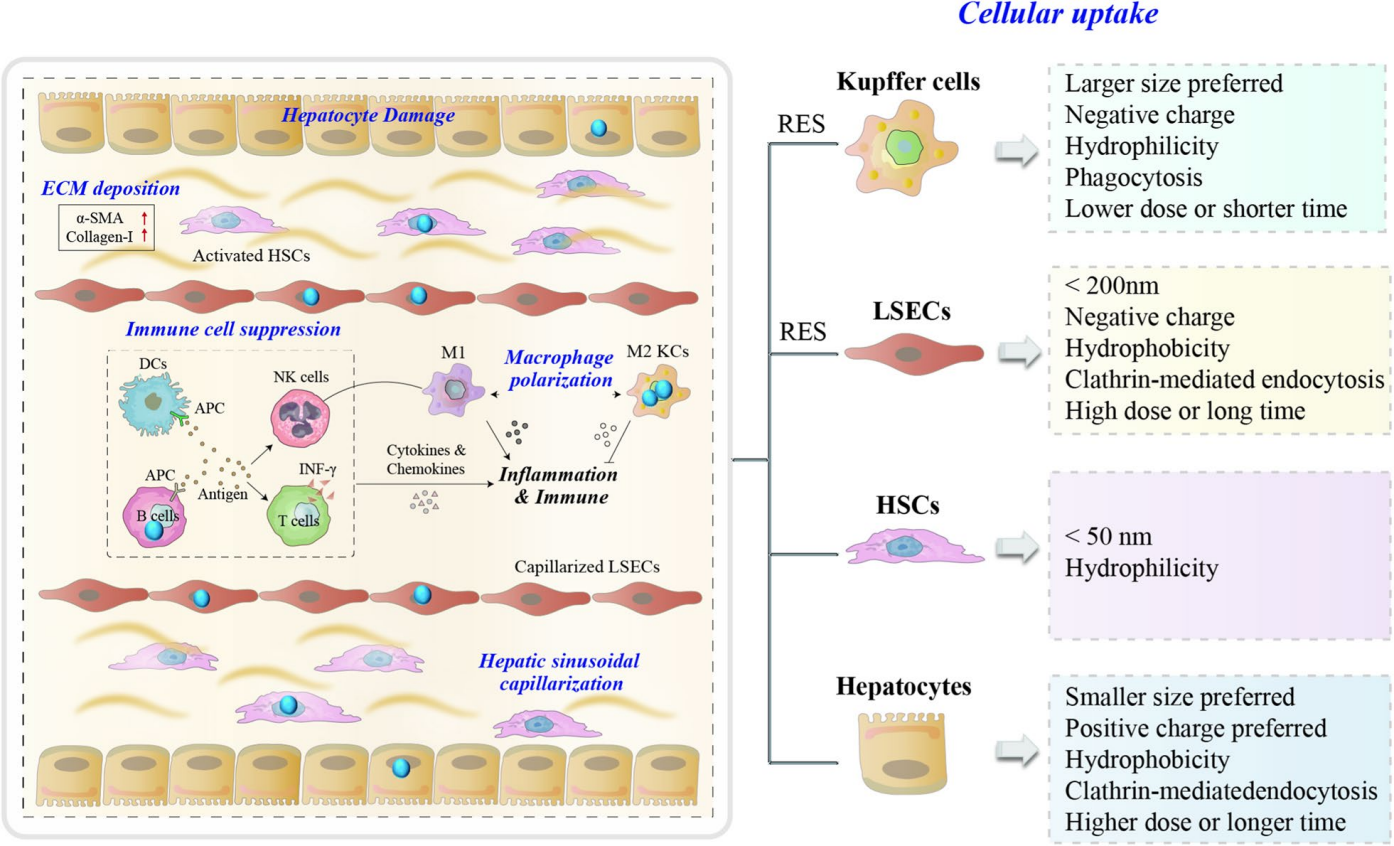
Interactions of NPs uptake and elimination in the liver during systematic circulation after NPs exposure

Although these NPs have a challenging and lengthy process, such as systemic circulation, drugs accumulation in the lesion, deep penetration, and intracellular release of drugs, the liver is one of the major aggregation organs for NPs, conventional NPs are recognized as foreign bodies, and rapidly captured by the reticuloendothelial system (RES) through the regulation of plasma proteins after administration, but the liver targeting NPs may serve as a beneficial nanomaterial for liver disease therapy [12], as shown in Fig. 2. As NPs move along the sinusoid, they will come into contact with sinusoidal endothelial cells, Kupffer cells (KCs), T cells, and DCs. Depending on their physicochemical properties, NPs have better access through fenestrae to enter the space of Disse and contact with hepatocytes. The smaller NPs may transcytose through the hepatocytes and enter the bile duct through bile canaliculi. Conventional NPs are captured by the RES. Larger size, negatively charged, or hydrophilic NPs are preferentially swallowed by KCs via phagocytosis; NPs less than 200 nm NP or with negative surface charge or hydrophobicity tend to be taken up by endothelial cells through clathrin-mediated endocytosis with a high exposure dose or long time. NPs less than 50 nm NP or hydrophilic NPs could be captured by stellate cells. Smaller NPs with positive surface charge or hydrophobic NPs are preferentially taken up by hepatocytes through clathrin-mediated endocytosis.

**Table 2:** Preclinical NPs for the treatment of liver fibrosis

NP type	Model	Target cell	Effect	Refer.
PEG/PVA NPs		MDDCs	Activating APCs and triggering the activation of CTL to produce long-term memory immunity	[123]
Fullerene-derived NPs		DCs	Th1 polarization response	[124]
PLGA NPs		DCs	Disrupting antigen processing and	[125]
GO		DCs	Disrupting cross-presentation	[126]
SPION		DCs	Reducing CD4 + T cell activation	[127]
SAS-NPs		moDCs	M2 macrophages to M1 phenotype and restored the activity of CD8 + T cells	[121]
Silica NPs		DCs	Generating pro-inflammatory cytokines through the P2X7R	[118, 122]
XL-MSNs		macrophages	Promoting M1 to M2 and inducing pyroptosis	[134, 135]
HA-PEI NPs		macrophages	Modulating the polarity of macrophages	[136, 137]
TiO2 anatase NPs		Macrophages, neutrophils	Inducing CD8 + T cell response initiation	[140]
TiO2, CeO2, and ZnO NPs		neutrophil	decreasing CD35 but increasing CD66b and CD63 expression	[145]
siVCAM-1 NPs		neutrophil	exerting an anti-inflammatory effect	[144]
SWCNTs		neutrophil	inhibiting both neutrophil migration and adhesion	[146]
Nanoparticle-bound NKT		NKT	activating T cell immune responses	[149]
USSN		T cell receptors	promoting T-cell activation, migration, and phenotypic transformation	[150]
lipid NPs		T-cell	anti-CD3-conjugated	[151]
liposomal PHA		T-cell	mediating T-cell activation	[152]
LNPs		CD4 + cells	mediating T-cell activation	[155]

Hence, we reviewed the latest advances in remodeling the hepatic fibrotic microenvironment with emerging monotherapies, with particular attention to remodeling immune regulation, metabolic reprogramming, ECM deposition, and hypoxia-induced vascular production in the hepatic fibrotic microenvironment. Finally, the nano-advantages and challenges of engineered NPs targeting APCs or directly targeting T cells for immunotherapy of liver fibrosis were highlighted in this review.

## Engineered NPs regulated immunosuppression-associated microenvironment

The immune system is a complex network including lymphoid organs, cells, and cytokines [13]. RES uptake and immune cell suppression are considered the main immune-related effects. In the liver, the RES, composed of LSECs and KCs, is the primary site of exposure to microbial antigens and plays a vital role in the uptake and clearance of soluble antigens from the hepatic sinusoids, serving as a guard against microbial invasion and maintenance of hepatic homeostasis [14]. Once fibrosis starts, LSECs change their phenotype (from fenestrations to capillaries) and then they open windows and the surface gradually shrinks to form an organized basement membrane, that is, "hepatic sinusoidal capillarization". In addition, LSECs serve as gatekeepers of the hepatic microenvironment and as platforms for innate or adaptive immune cells to stay in the hepatic sinusoidal microenvironment. This is important for maintaining systemic immune homeostasis [15].

### LSECs-related immunosuppression-associated microenvironment

In the innate immune response, LSECs have an effective function by directly participating in the suppression of activated CD4 + T cells [16], or by expressing major histocompatibility complex (MHC) class I and II molecules presented to CD8 + T cells and promoting the activation of regulatory T cells (Tregs) [17]. In addition, LSECs express a variety of pattern recognition receptors including the toll-like receptor (TLR) family, scavenger receptors (SR-A, SR-B, and SR-H), and mannose receptors (MR) that produce inhibitory acquired immune responses. LSECs regulate adaptive immune responses directly by presenting antigens to T cells and also regulate natural killer T cells (NKT cells) by expressing CXCL16, and the cell surface ligand for CXCR6 [18]. Hepatic macrophages account for 90% of the total macrophages in the human body, and they are very plastic and adapt their phenotype according to signals derived from the hepatic fibrotic microenvironment [19]. They are divided into infiltrating macrophages and liver-resident macrophages[20]. Liver-resident macrophages, called KCs are self-renewed, resident, and non-migratory [19]. Liver injury triggers KCs activation, leading to inflammatory cytokine and chemokine release. This fosters the infiltration of monocytes into the liver, which gives rise to the large number of inflammatory monocyte-derived macrophages [21] Moreover, KCs promote T cell-mediated hepatitis development by producing CXCL10 and limiting the permeability of hepatic LSECs [22]. The macrophage pool of the liver can be rapidly expanded by infiltrating phagocytes that mainly originate from peripheral blood marrow/monocyte-derived macrophages [21], a few from peritoneal macrophages [23], and splenic macrophages [24]. In mice, two major populations of circulating monocytes exist Ly-6C high (Ly-6C^hi^) and Ly-6C low (Ly-6C^lo^) expressing monocytes. Whereas the Ly-6C^hi^ monocytes express inflammatory chemokine receptors (like CCR2), pattern-recognition receptors, and cytokines [25]. The bone marrow is the primary source of the (relatively immature) Ly-6C^hi^ monocytes [26], whereas the spleen serves as a reservoir for Ly-6C^lo^ monocytes, the production of TNF-α, IL-6, and IL-10 increased significantly in hepatic splenic macrophages and migrate from bone marrow to the liver via the spleen [27]. As a consequence of tissue injury, KCs and other liver cells (HSCs, hepatocytes) secrete chemokines like CCL2 that provoke the massive infiltration of Ly6C^hi^ monocytes into the injured liver [28]. This provides a rapid and transient mechanism to expand the macrophage pool in the liver by inflammation-prone phagocytes. Together, they form a "profibrogenic environment" in which immune homeostasis is disrupted, which may explain the refractory nature of immunotherapy for liver fibrosis, as shown in Table 1.

**Fig. 3:**
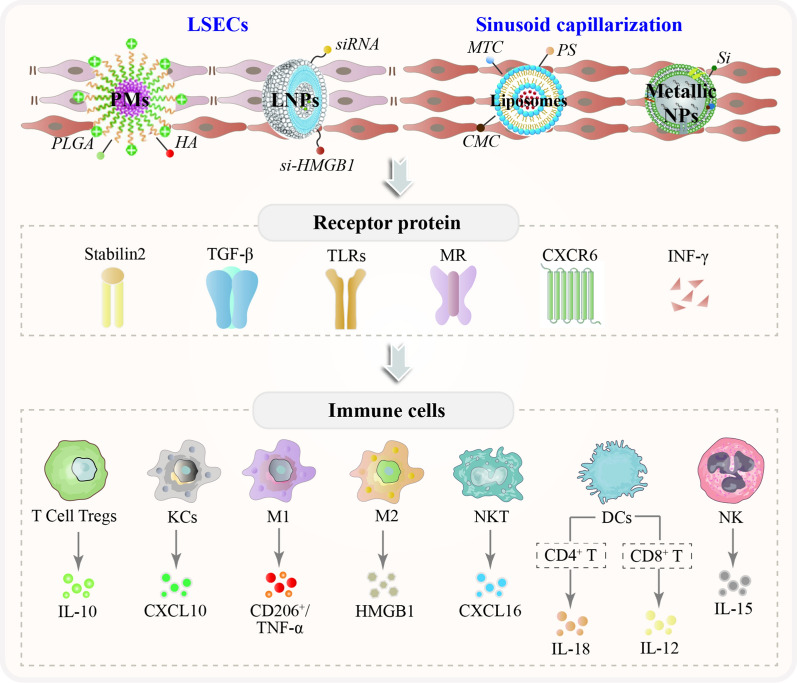
The NPs in LSECs capillarization-mediated immunosuppression-associated microenvironment of liver fibrosis

Polymeric micelles (PMs) as drug delivery vehicles have specific targeting and high stability in vivo as illustrated in Fig. 3. Scavenger receptors form a superfamily of membrane-bound receptors, and in vitro studies have shown that anionic NPs modified with scavenger receptor stabilizer-2 (stab2) receptor ligands target naturally tolerant LSECs and generate Tregs, thereby suppressing antigen-specific immune responses [29] and which also leads to selective deletion of single blood vessels in zebrafish embryos, with important clearance of lipoprotein B-containing lipoproteins in zebrafish [30]. Hyaluronic acid (HA) is a naturally occurring ligand and encapsulated micelles that show sustained drug release and low cytotoxicity for targeting LSECs, with over 90% of HA in the blood that is absorbed and metabolized by LSECs [31] LSEC-targeting and fenestrae-repairing nanoparticles (named HA-NPs/SMV) rapidly released SMV and exerted a fenestrae-repairing function, providing an antifibrotic therapeutic regimen. [32]. Studies have shown that coating positively charged PMs consisting of poly(L-lysine)-block-poly(L-lactide) (PLys-b-PLLA) AB diblock copolymers with anionic HA by polyion complex (PIC) formation target specific interaction between LSECs and KCs, thereby increasing the toxic T-lymphocyte/Treg cell ratio and then cause immune inflammation [33]. Poly (lactic-co-glycolic acid) (PLGA) has been used in many long-acting drug formulations approved by the US Food and Drug Administration (FDA) [34]. NPs decorated with stab ligands and PLGA target naturally tolerant LSECs are capable of producing Tregs to inhibit antigen-specific immune responses [33].

### Macrophages-related immunosuppression-associated microenvironment

**Fig. 4:**
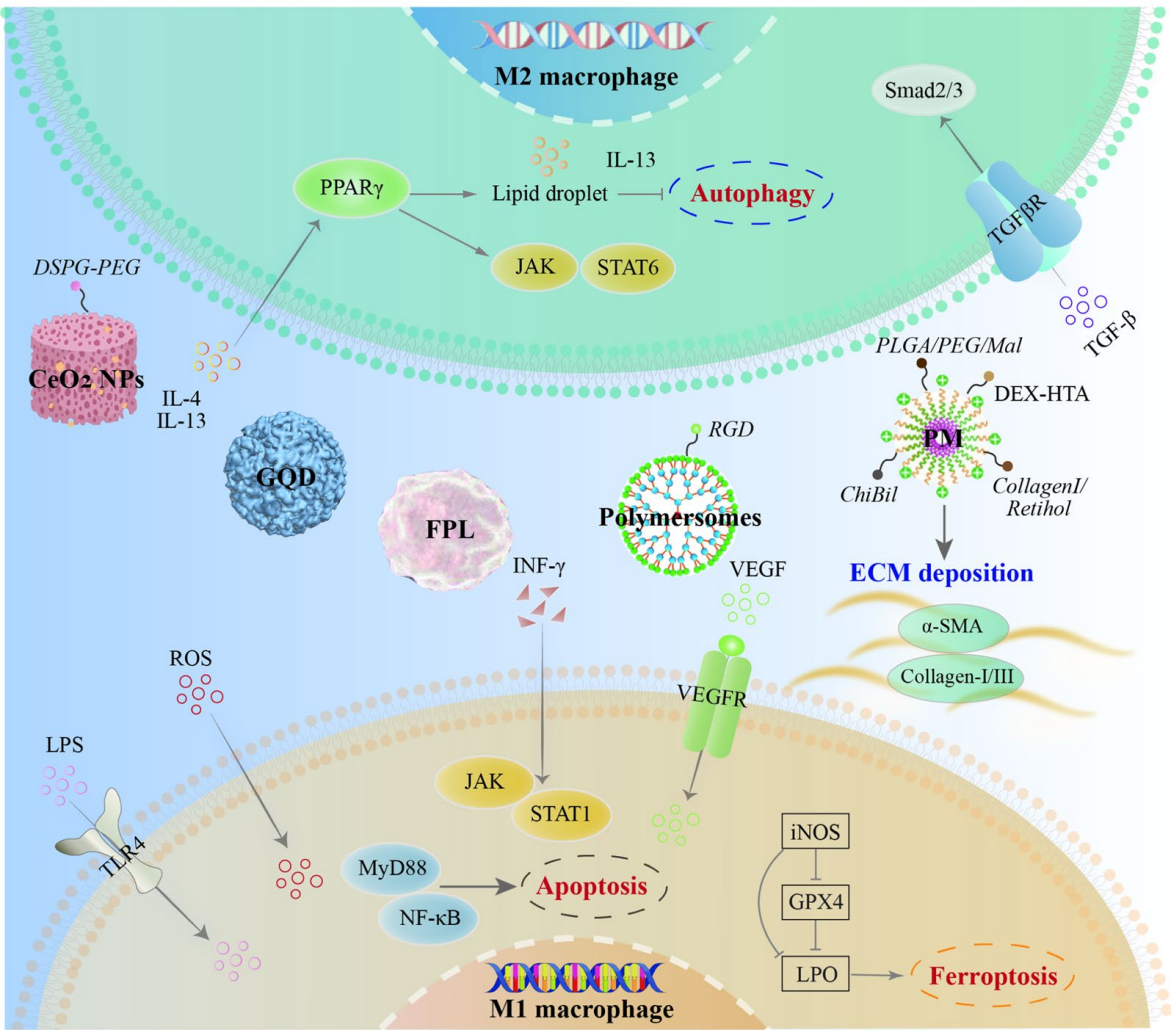
The NPs in macrophages polarization-mediated immunosuppression-associated microenvironment of liver fibrosis

Activated macrophages can release cytokines, which can affect the adaptive immune response [35]. It was illustrated in Fig. 4 that MR directly promotes the pro-inflammatory activation of macrophages and triggers inflammation [36]. Mannose-modified albumin NPs have the potential to deliver TGFβ-siRNA to CD206 + macrophages as an anti-fibrotic strategy [37]. A stable nucleic acid–lipid particle delivery system of mannose-modified HMGB1-siRNA (mLNP-siHMGB1) targets hepatic macrophages via mannose receptor-mediated targeting, thereby silencing HMGB1 protein expression and inhibits the activation of HSCs for the treatment of liver fibrosis [38]. Mannose-modified trimethyl chitosan-cysteine (MTC)-coupled NPs as efficient polymeric carriers for oral TNF-α siRNA, and a dextran-based siRNA carrier system, BG34-10-Re-I/siRNA, was also developed for macrophage-targeted siRNA delivery [39]. Carboxymethyl chitosan (CMC) is a promising drug-release polymeric carrier with biocompatible, biodegradable, and easily accessible features. CMC reduces host immune response in an experimental mouse model of CCl4-induced chronic liver injury [40]. Phosphatidylserine (PS) is a phospholipid with a negatively charged head that is normally found in the inner leaflet of the cell membrane. PS-containing NPs are commonly used to mimic apoptotic cells and can specifically modulate macrophage function and enhance the targeting ability of macrophages [41], PS-modified nanostructured lipid carriers were further designed in some previous studies to improve hepatic delivery efficiency and its bioavailability, thereby reducing liver fibrosis and collagen fiber deposition in vivo [42]. The scaffolds crosslinked with nano-graphene oxide show high resistance to enzymatic degradation via direct inhibition of MMPs activity and increased M2-like macrophage polarization, which reduces graft-elicited inflammation (Table 2). Overall, nano-graphene oxide offers an alternative for donor organs [43].

## Engineered NPs regulated metabolic reprogramming-associated microenvironment

Liver injury stimulates the transdifferentiation of quiescent hepatic stellate cell HSCs to proliferative, migratory, and fibrotic myofibroblastic (MFs) [44]. The short-term accumulation of MFs is beneficial for liver regeneration, however, long-term excessive accumulation results in progressive fibrosis, repair defects, and increase risk and mortality from cirrhosis and liver cancer [45]. The acquisition of MFs phenotype is energy-intensive, whereas reprogramming of HSCs in metabolism is similar to that of highly proliferative cancer cells, and orchestrating the reprogramming of HSCs is a novel therapeutic target for fibrosis therapy [46, 47], including.

1. Enhanced aerobic glycolysis [48]. Elevation in glycolysis is related to an increase in glucose transporter proteins, including GLUT1, which is highly overexpressed in cancer cells [49] In addition, an increase in glycolysis is accompanied by a shunt of central carbon metabolites from the citric acid cycle, including increased expression of pyruvate dehydrogenase kinase 3 (PDK3), which promotes lactate production. A similar phenomenon is called the Warburg effect in cancer cells [50]. Furthermore, pyruvate kinase M2 (PKM2) represents a unique link between aHSCs and cancer cells that promote aerobic glycolysis [51].
2. Upregulation of glutamine catabolism [52]. Glutamine metabolism has been identified as an additional source of ATP in HSCs. Liver samples from patients with nonalcoholic steatohepatitis (NASH) and advanced fibrosis [53] and a mouse model of liver fibrosis [54] have shown that the proliferation of HSCs is heavily dependent on glutamine metabolism [52]. Metabotropic glutamate receptor-5 (mGluR5) production of 2-arachidonic acid glycerol (2-AG) in HSCs activates hepatocyte cannabinoid receptor-1 (CB1R)-mediated neoadipogenesis [55].
3. Fatty acid catabolism [56]. Compared to the resting HSCs, activated HSCs have abundant mitochondria, and the requirement to maintain the phenotype of MFs still needs the energetic contribution of oxidative phosphorylation (OXPHOS). It has been shown that mitochondrial uncoupling inhibits the activation of HSCs in vitro, despite increased glycolysis [57]. The metabolism of lipid droplets that provide fatty acids for mitochondrial β-oxidation (FAO) plays an important role in lipid metabolic pathways [58]. Similarly, in cancer, FAO survives oxidative stress and nutrient deprivation [56].

### HSCs-related metabolic reprogramming-associated microenvironment

**Fig. 5:**
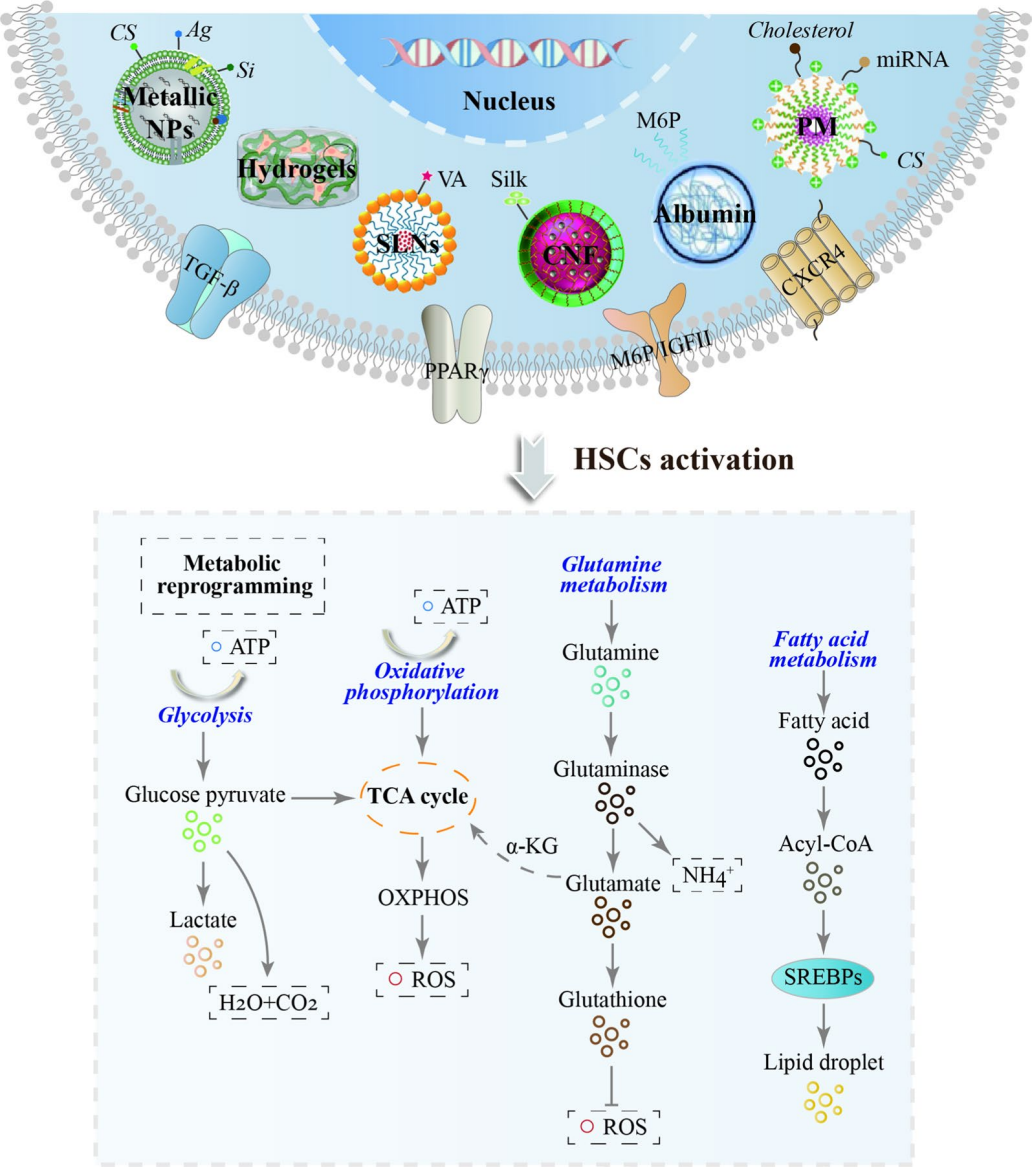
The NPs in HSCs activation-mediated metabolic reprogramming-associated microenvironment of liver fibrosis

It was illustrated in Fig. 5 that the HSCs expressed the mannose-6-phosphate/insulin-like growth factor II (M6P/IGFII) receptor, and M6P-modified albumin activates HSCs in the fibrotic liver, blocks glutamine (GLN) catabolism by mediating the Hedgehog signaling pathway Glutaminases including GLS (glutaminase), aspartate aminotransferase (GOT1) and glutamate dehydrogenase (GLUD1) and inhibits myofibrillar activity [59]. GLN metabolism is an important component of metabolic reprogramming. GLN can be converted to α-ketoglutarate (α-KG) to provide carbon for the TCA cycle, or to other NEAAs via transaminases (GOT1 and GOT2). GLN can also be converted to glutamate and pyrroline-5-carboxylic acid (P5C) to stimulate collagen biosynthesis [60]. HSCs have multiple vitamin A (VA)-rich lipid droplets in the cytoplasm, which are the primary sites for retinoid derivatives storage in vivo. A study has synthesized a novel VA-Myrj52 ester conjugated solid lipid NPs (VA-SLNs) using all-trans retinoic acid and hydrophilic emulsifier (Myrj52) as targeting agents to effectively reduce peroxisome proliferator-activated receptor γ (PPARγ)/SREBPs-mediated lipid accumulation [61]. SREBPs are highly expressed to promote tumor growth, especially in the regulation of lipid metabolism [62]. In addition, chondroitin sulfate PMs target HSCs in liver fibrosis [63]. Green biosynthesis of NPs using reduced metabolites of microbial and plant-derived products is a better strategy for achieving inexpensive products that are less harmful to health and the environment compared to artificial physical or chemically manufactured NPs [64]. TGFβ signaling is involved in stimulating glycolysis and mitochondrial respiration. Inhibitory effect of curcumin/chitosan-coated green silver NPs directly bound to the fibrogenic protein TGF-β [65]. Mouse livers were decellularized to form liver hydrogels as an injectable biomaterial in the liver, which blocked the TGF-β1/Smad pathway to reduce fibrosis [66] (Table 2).

### Immune cells related metabolic reprogramming-associated microenvironment

Regarding macrophages and KCs, restriction of their glucose and glutamine supply inhibits their secretory function [67]. Conversely, altered lipid metabolism causes KCs to accumulate cytotoxic lipids to enhance the proinflammatory phenotype [68]. The enhanced glycolytic activity, altered tricarboxylic acid cycle, and reduced ATP production in the macrophages under 100–125 nm diameter NPs made of silk, poly(lactic-ethanolic acid), or silicon consistent with a pro-inflammatory phenotype [69]. PEGylation of cellulose nanofiber (CNF) reduces macrophage-initiated inflammatory and metabolic responses, including increased glycolysis and reprogramming of the tricarboxylic acid cycle and the creatine kinase/phosphocreatine pathway [70]. Energy metabolism is also involved in the body's immune network. It has been shown that metabolic reprogramming between immune cells and cancer-associated fibroblasts (CAFs) indicated the immune status of tumors [71]. aCD3/F/AN-induced lipid metabolic reprogramming specifically activates T cells [72]. A recent study synthesizes cholesterol-modified polymeric CXCR4 inhibitor (Chol-PCX) in the form of Chol-PCX/miRNA NPs and CXCL12/CXCR4 axis disrupts the lipid metabolic network of T cells for the amplified treatment of liver fibrosis [73, 74], suggesting that nanotechnology-enabled T cell lipid metabolic reprogramming has the potential to be a new paradigm for immunometabolic therapy.

## Engineered NPs regulated hypoxia-associated microenvironment

Activated HSCs lose the ability to store retinol, then they begin to proliferate and produce pro-fibrotic cytokines such asα-smooth muscle actin (α-SMA), type I, and type III collagens [44]. Whereas collagen fibers between adjacent hepatic blood sinusoids contact each other and encircle hepatocytes in a grid pattern, which weakens oxygen exchange between hepatic sinusoids and hepatocytes, therefore leads to hypoxia in the microenvironment of liver fibrosis [75]. In addition, abnormal immune inflammation leads to the release of inflammatory factors, chemokines, ROS, adipokines, and pro-angiogenic mediators [76]. Among them, intracellular NO and ROS reactions are significantly increased in extracellular matrix (ECM) subjected to peroxynitrite (ONOO-)-mediated oxidization, and MMP-2 activity, directly mediated by S-glutathionylation of its cysteine residues in the presence of ONOO(-) and by phosphorylation of its serine and threonine residues [77], as well as increased fibrosis [78]. Excessive deposition of fibrillated collagen (mainly collagen I), the main collagen of ECM, in the Disse space would greatly impede the delivery of HSCs by nanoformulations [79]. Collagenase-I decoration promises an efficient nano-drug delivery system for HSCs-targeted Collagenase-I-decorated co-delivery micelles to enhance extracellular matrix degradation and HSCs-targeted therapy [80]. A polymeric micelle co-decorated with collagenase I and retinol was prepared, and an ECM-penetrating nano drill micelle with nanoscale and HSCs-targeting capabilities, based on poly(lactic-co-glycolic)-b-poly (ethylene glycol)-maleimide (PLGA-PEG-Mal) (polymeric micelle named monophosphate (CRM)) for the treatment of liver fibrosis. Upon encountering the collagen I barrier, CRM effectively degrades the pericellular collagen I [79]. Retinol binds to low molecular weight polyethyleneimine (PEI), which further binds to nucleotides (RcP) to form NPs, allowing RcP carriers (RAP) containing antisense oligonucleotides (ASO) to enter HSCs directly [81]. This nanoparticle system actively recruits plasma proteins, particularly retinol-binding protein 4 (RBP4), which forms a corona on the surface and effectively inhibits collagenase-I expression, thereby ameliorating liver fibrosis. In addition, Oxymatrine (OM) modified with a nanosystem of cyclic RGD peptide-modified poly(ethylene glycol)-b-poly(ε-caprolactone) (PEG-b-PCL) was previously reported to target HSCs and reduce serum levels of PC-III and IV-C, which aggregate and inhibit the proliferation of HSCs [82]. Studies have confirmed that integrins and adhesion proteins in the extracellular matrix, such as type VI collagen and fibronectin, and cyclic arginine aspartate (RGD) peptide C*GRGDSPC* (* indicates cyclized cysteine residues) are ligands for type VI collagen receptors that can effectively target drug delivery to HSCs [83].

Antioxidant nano enzymes are artificial enzymes based on nanomaterials that can modulate the activity of multiple antioxidant enzymes and target the liver, and hence can be used as a novel therapeutic option. ROS-mediated oxidative stress exacerbates mitochondrial dysfunction, which releases mitochondrial DAMPs and disrupts oxygen homeostasis, and further exacerbates the microenvironment of liver fibrosis [84]. Antioxidant nano enzymes for liver injury therapy are mainly based on cerium oxide, melanin-like carbon (fullerenes, graphene, and other carbon nanomaterials), and other nanomaterials such as selenium and MXene. cerium oxide nanoparticles (CeO2 NPs) represent an extensively studied type of multi-antioxidant with potent nano enzyme activity, including GSH and CAT mimicking properties [85]. The CAT-mimetic activity of DSPE-PEG-CeO2 NPs converts harmful H_2_O_2_ to O_2_ to alleviate the hypoxic environment and reduce inflammation [86]. In addition, carbon and graphene-based nanomaterials have two-dimensional conjugated structural domains that scavenge ROS. the ROS quenching potential of graphene arises from surface defects and unpaired electrons, and graphene quantum dots (GQD) inhibit lipid peroxidation, apoptosis, and autophagy in Concanavalin A (ConA)-induced hepatitis in mice [87]. Fulleropyrrolidine (FPL), on the other hand, has a three-dimensional conjugated structural domain and is an effective antioxidant in *vivo* [88]. Furthermore, the stimuli-responsive transformation in the crosslink nano-delivery strategy is emerging. A chitosan-bilirubin micelle (ChiBil) carrying losartan attenuates iron death due to iron-catalyzed lipid peroxide (LPO) accumulation in liver fibrosis [89]. ROS-responsive DEX/HA-TK-ART PMs induce M2 macrophages via HIF-1α/NF-κB signaling cascade [90]. In addition, in hepatocellular carcinoma, a two-pronged approach of T-SPNAPt/NO-enhanced damage-blocking repair, a reactive nitrogen species (RNS)-generating system, was developed to achieve efficient treatment [91] (Table 2).

## Engineered NPs regulated angiogenesis-associated microenvironment

Hypoxia induces sustained production of pro-angiogenic factors [92], especially VEGF, which survives obstructive angiogenesis and promotes the secretion of TGF-β and IL-10, thereby enhancing immune tolerance. In addition, capillarization of LSECs reduces the bidirectional transport of substances in the hepatocyte and perisinusoidal space, and the hepatic sinusoids are the main sites for the regulation of blood flow, and the reduction in the size and number of fenestrations cause a disruption of the vascular structure, the deposition of ECM causes a significant increase in the intrahepatic vascular resistance (IHVR) and the development of portal hypertension. In addition, hypoxia-dependent or non-dependent pathological angiogenesis abnormalities also exacerbate the hepatic microenvironment disorder, constituting a vicious cycle of hepatocyte injury, eventually leading to liver failure or cirrhosis [93].

**Fig. 6:**
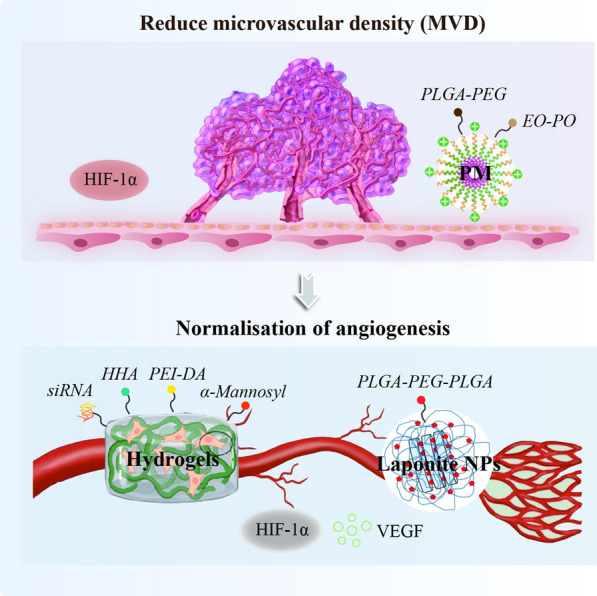
The NPs in hypoxia/angiogenesis-associated microenvironment of liver fibrosis

Figure 6 showed that altered ratios between vasodilators and vasoconstrictors produce vascular shunts and functional abnormalities that lead to disturbances in the hepatic microenvironment. LSECs regulate vascular tone by producing nitric oxide (NO). Their dysfunction is mainly manifested by impaired eNOS activation and reduced the levels of hepatic vasodilator NO synthesis [94]. Statins inhibit the activity of HSCs and KCs, thereby decreasing intrahepatic vascular tone and portal hypertension [95]. Self-assembled PMs based on Pluronic® amphiphilic copolymers consisting of ethylene oxide (EO) and propylene oxide (PO) chains arranged in a triblock structure (EOa-POB-EOa) are biodegradable and biocompatible. Polymer micelles loaded with simvastatin accumulate in LSECs, avoiding the hepatotoxicity and side effects caused by conventional simvastatin [96]. To some extent, hepatocytes and HSCs regulate the phenotype of LSECs through paracrine secretion of VEGF, Ang-1 or Ang-2, PDGF-BB, and Hedgehog ligands, which may drive fibrogenesis and fibrotic spacer formation mediating hypoxia-dependent angiogenesis. Sorafenib is a bifunctional tyrosine kinase inhibitor that blocks Raf/MEK/ERK pathway and the VEGFR/PDGFR. Past studies have shown that sorafenib treatment inhibits angiogenesis, thereby improving liver fibrosis [97]. PLGA has hydrophobic properties and biocompatibility [98]. PEG-PLGA reduces size polydispersity and increases the stability of NPs in circulation [99]. It was shown that PEG-PLGA has profound anti-fibrotic activity in a CCl4-induced fibrosis model, prolonged the circulation of modafinil, significantly constricted abnormal blood vessels, reduced microvascular density (MVD), and normalized blood vessels in the fibrotic liver.

In addition, local stimulation of angiogenesis is an important way to enhance ischemic or repair injured tissue. Deoxycholic acid-modified polyethylene glycol polymer conjugate (PEI-DA) conjugates deliver LNA-92a in vitro and in vivo to improve angiogenesis. Naturally derived and synthetic hydrogels can promote post-ischemic tissue remodeling [100]. Furthermore, local delivery of RNA interference (RNAi)-based therapies via hydrogel-loaded PEI-DA polymorphic NPs appears to be a safe and effective approach for different therapeutic targets [101]. Nowadays, it is generally accepted that M1 macrophages, as pro-inflammatory macrophages, mainly exert antigen-presenting function, and have pro-inflammatory. They are mainly induced by lipopolysaccharide (LPS) and interferon-γ (IFN-γ) [20]. Toll-like receptor 4 (TLR-4) is an innate immune receptor that is the main receptor of LPS [102]. LPS binds to TLR4 to activate nuclear factor-κB (NF-κB) through the myeloid differentiation factor 88 (MyD88)-dependent pathways [103]. IFN-γ binds to its receptor and activates JAK, thus inducing the phosphorylation of STAT1, which leads to the polarization of macrophages to M1 [104]. M2 macrophages are known as anti-inflammatory macrophages, producing anti-inflammatory factors, such as IL-10, transforming growth factor-β (TGF-β), and arginase 1 (Arg1) [105]. TGF-β/Smads signaling pathway in promoting M2 macrophage polarization [106]. Whereas PPARγ is involved in the process of M2 macrophage polarization induced by interleukin (IL)-4 and IL-13 [20]. JAK/STAT6 is an important pathway by which IL-4 inhibits M1 and induces M2 polarization [107]. M2 macrophages overexpress the mannose receptor CD206, targeting these cells to the immunostimulatory and antifibrotic M1 phenotype through CD206 and repolarization of macrophages by RNA interference represents an attractive therapeutic approach. Studies have designed nanosized hydrogel particles with mannose residues on the surface to deliver siRNA more efficiently to M2-polarized macrophages [108]. Double cross-linked hyaluronic acid (HHA) hydrogel for supply and modulation of the M2 phenotype of macrophages to synergistically improve immunocompromised and impaired angiogenesis [109]. In addition, the use of injectable, biodegradable nanocomposite hydrogel scaffold injectable, biocompatible, biodegradable nanocomposite gel composed of poly(dl-lactide-co-collide)-b-polyethylene glycol-b-poly(dl-lactide-co-collide) (PLGA-PEG-PLGA) copolymer and clay NPs laponite, the interaction between DFO and LAPONITE enhances HIF-1α and also improves VEGF expression, thereby accelerating neovascularization [110]. Proteins are too unstable to ensure their biological effects, whereas laponite is an inorganic, stratified granular material that occurs as a natural product of rock [111], which is highly adsorbent to biomolecules [112] and synthetic nanoclays. For example, laponite™ is safe and well tolerated even at high doses and spontaneously forms irreversible gels when in contact with blood proteins and ions [113]. Furthermore, Decellularized tissue hydrogels retain intrinsic molecules and showed greater biocompatibility and bioactivity compared to synthetic hydrogels [114] (Table 2).

## Engineered NPs targeted T Cells in liver fibrotic immunotherapy

**Fig. 7:**
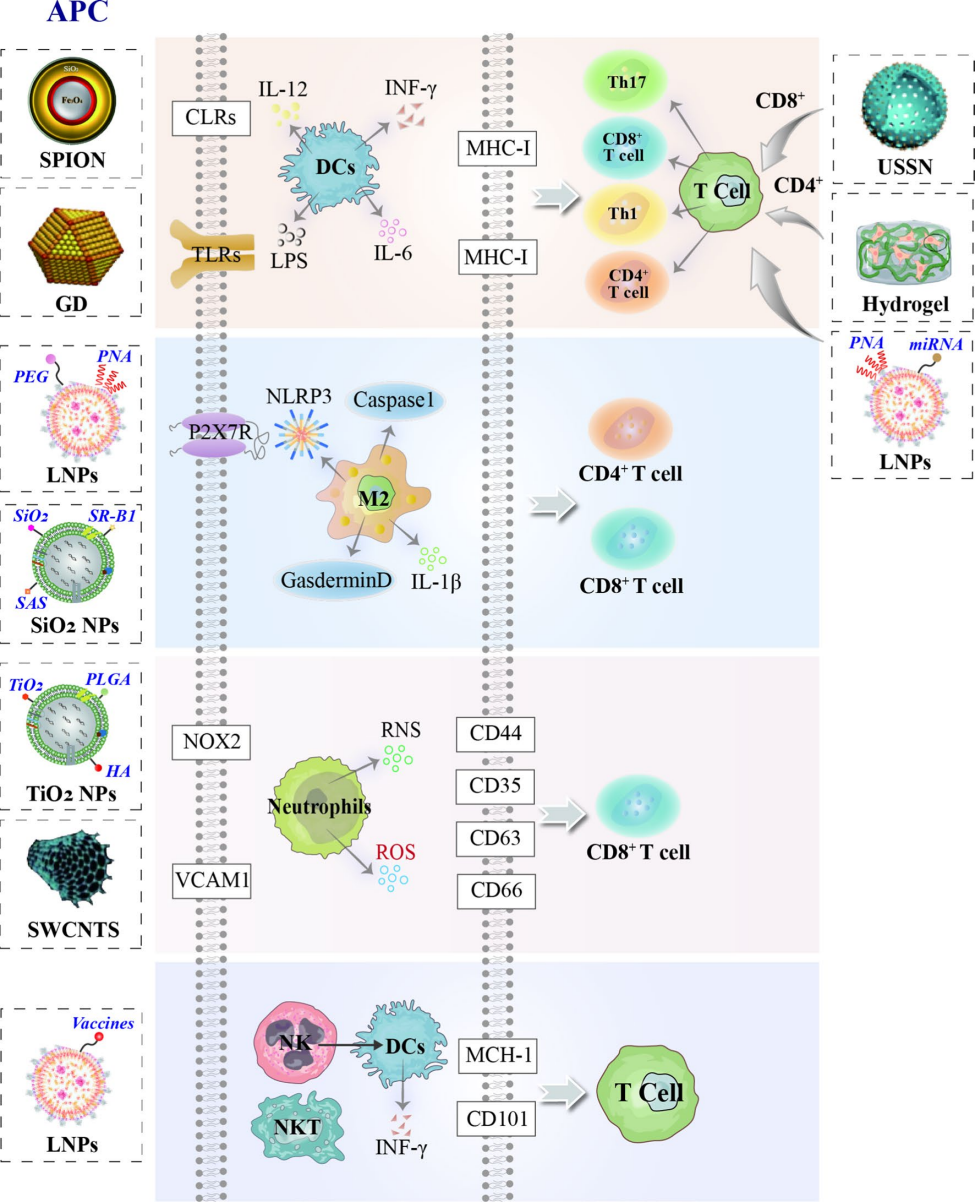
The NPs targeted specialty APCs/T cells-associated an innate/acquired immune-like therapy in hepatic fibrotic microenvironment

Improving the immune infiltration in the hepatic fibrosis microenvironment is an effective strategy for patients with liver fibrosis [115]. A comprehensive analysis of infiltrating immune cells in the liver will elucidate the mechanisms of liver fibrosis-immune evasion, thereby providing opportunities for the development of new therapeutic strategies. The interaction between NPs and various components of the immune system has become an interesting research area in biology and nanotechnology. The immune system fights against foreign microorganisms and interacts with engineered NPs. Design nanomedicine products such as drug delivery systems enable the immune system to effectively recognize and utilize NPs that can not only modulate the immune response but also evade immune surveillance. Hence, they are more effective in exhibiting their therapeutic potential. Therefore, assessing how NPs interact with the immune defense system is a key issue in the current approach to safely design nanotechnology and nanomedicine products to determine the safety and hazard of NPs for human and environmental health, and the opportunity and challenge to integrate delivery technologies into immunotherapy for liver fibrosis. The engineered NPs provide a viable platform for each stage of therapeutic T-cell response. Below we discuss the various methods for generating and maintaining antigen-specific T-cell responses to immunotherapy and how nanomaterials can be used to enhance these processes, as shown in Fig. 7.

### Targeted specialty APCs, an innate immune-like therapy

Specialized APCs are effective strategies for T-cell therapy through efficiently presenting antigens, providing upstream opportunities for immune regulation, and effectively directing T cell's behavior.

NPs are recognized as foreign particles via PAMPs and DAMPS classical pathways or intracellular LPS of Gram-negative bacteria that further link to TLRs expressed. DCs are the most efficient APCs that initiate CD4 + or CD8 + T cells through antigen cross-presentation to MHC-II or MCH-I, respectively and its different isoforms produce functional IL-18, which enhances IL-12-dependent Th1 immune responses [116] and thereby produce high levels of type I interferon, which promotes maturation and polarization of macrophages, hence promoting immunostimulatory hepatic fibrosis microenvironment.

The size, shape, and surface chemistry of NPs have been shown to interact with multiple pathways to activate immune pathways. In particular, systematic dual targeting of C-type lectin receptors (CLRs) DC-SIGN and TLR7 of DCs using mannosylated antigens [117]. Silica NPs activate purinergic signaling through the P2X7 receptor (P2X7R) of DCs, generating pro-inflammatory cytokines [118]. Activation of the P2X7R induces the formation of the nucleotide-binding domain, the leucine-rich family, the pyridine-containing domain 3 (NLRP3) inflammasome, and the activation of inflammatory caspase-1. Inflammasomes require oligomerization to oligomerize and activate pro-caspase 1 to caspase 1 and then convert pro-IL-1β to IL-1β to activate NLRP3 which has been reported to respond to a variety of different stimuli, including silica and aluminum crystals, and asbestos [119]. SR-B1 is a silica receptor associated with typical inflammasome activation [120]. Synthetic amorphous silica nanoparticles (SAS-NPs) induce the maturation of functional moDCs [121]. Metal NPs impairs the maturation and heterogeneous stimulation ability of LPS-induced DCs, and the effect of 10 nm is more prominent compared to 50 nm [122]. However, being coated with polyethylene glycol (PEG) and polyvinyl alcohol (PVA) to make their surfaces positively or negatively charged, there is no difference in the uptake of monocyte-derived MDDCs, both of which can effectively activate APCs and trigger the activation of CTL to produce long-term memory immunity [123]. Fullerene-derived NPs of 10–60 nm induces functional DCs to stimulate a tilt toward a Th1 polarization response [124]. DCs exposed to cationic, neutral, or anionic PLGA NPs do not produce cytotoxicity[125]. graphene oxide (GO) [126] and superparamagnetic iron oxide pellets (SPION) [127] disrupted antigen processing and cross-presentation as well as reduced CD4 + T cell activation.

NPs regulate macrophage polarization and reprogramming with different chemical compositions, sizes, and surface modification, which is a promising immunotherapeutic strategy. For example, metal oxide NPs (for example. Ag, ZnO, TiO2) induce M1 inflammatory cytokines in a dose-dependent manner [128, 129].In addition, Au NPs are more effective than Ag NPs related to particle size [130]. Moreover, NPs not only induce polarization of macrophages but also reprogram them [131] SPION, glycocalyx-mimicking [132] and carbosilane dendrimer [133] induced a shift of M2 macrophages to M1 phenotype and restored the activity of CD8 + T cells, resulting in an immunological memory effect. Mesoporous silica NPs (XL-MSNs) with extra-large 30 nm pores deliver cytokines polarized by macrophages in vivo [134] and induced pyroptosis [135]. Similarly, the transition from M1 to M2 can be mediated by hyaluronic acid-poly(ethyleneimine) (HA-PEI) NPs [136] and CD44 targets efficiency in hyaluronic acid-poly(lactic acid) (HA-PLA) NPs to modulate the polarity of macrophages [137].

Neutrophils have a short life cycle of 12 h. They are very responsive and are the pioneers of innate immunity [138], Neutrophil's defense against apoptotic hepatocytes, and can influence the activation of different types of leukocyte types, including NK cells, B cells, and DCs. Endocytosis is the main mechanism of nanoparticle uptake by neutrophils. Respiratory burst is one of the endocytosis, which is an oxygen-dependent process that mediates immunosuppression [139]. Titanium dioxide (TiO2) anatase NPs have activated immune cells, including macrophages and neutrophils, leading to the production of ROS, infiltration of exogenous antigens into the cytoplasm, and the presentation by MHC-1 molecules thereby inducing CD8 + T cell response initiation [140]. Some ROS-responsive functional groups, such as peroxy ester groups, have been integrated into the structure of nano drugs. PLGA, modified by HA through peroxy ester bonding to PEG, can target CD44 cells to promote uptake by immune cells to ensure the controlled release of antigens, thereby facilitating the uptake of antigens [141]. In addition, ROS-triggered nanoparticle-based antigen delivery systems for promoting vaccine-induced immune responses are also under development [142]. PAA-coated and uncoated ION triggered an oxidative burst in the neutrophils in an NADPH oxidase-dependent manner, thereby promoting superoxide anion production by the NADPH oxidase complex and mediating lipid peroxidation. Activation of the NADPH oxidase 2 (NOX 2) complex enhances the cross-presentation of APC [143]. In addition, co-delivery of DXM with VCAM-1 siRNA (siVCAM-1) inhibits both neutrophil migration and adhesion, hence exerting an anti-inflammatory effect [144]. NPs could act as regulators of human neutrophil degranulation, with TiO2, CeO2, and ZnO NPs causing a slight decrease in the expression of CD35 however, it increases the expression of CD66b and CD63 [145]. Adsorption of plasma proteins on single-walled carbon nanotubes (SWCNTs) reduces cytotoxicity and modulates neutrophil activity [146]. NK cells act to specifically kill senescent HSCs and induce cell cycle arrest and apoptosis in HSCs through the release of IFNγ, enhanced by IL-15 [147]. NK and NKT do not require antigen and histocompatibility complex (MHC) activation, in addition to promoting APC maturation, and hence activate T cell immune responses by secreting INF-γ [148] Nanoparticle-bound NKT cellular ligands are immune adjuvant recognizing glycolipid antigens presented by MHCI/CD1d which are potentially relevant in the formulation of effective antiviral vaccines capable of eliciting activation of antigen-specific cell-mediated and humoral immune responses [149].

### Targeted T cells, an acquired immune-like therapy

APCs indirectly target T cells, whereas, NPs directly stimulate T lymphocytes, making it an ideal option.Ultrasmall silica NPs (USSN) (<10 nm in diameter) binds to T cell receptors and CD3 to induce the activation of T cell [150]. Situ T-cell transfection with anti-CD3-conjugated lipid NPs promotes T-cell activation, migration, and phenotypic transformation [151]. PEGylated liposomes, consisting of DPPC and cholesterol, can efficiently load PHA. These liposomes confer powerful T-cell activation *in vitro* and *in vitro*. Compared to soluble PHA, liposomal PHA formulations *in vivo* mediate T-cell activation with no-toxic [152]. The binding and uptake of CD4+ and CD8+ T cells were compared for amino-functionalized polystyrene beads (63-121 nm in diameter) [153]. Modulation of CD4+ T cells by immunomodulation can alter the course of development of autoimmunity and immunodeficiency. Antibody conjugation improves the uptake efficiency of CD4+ T-cell of nano gels [154]. T cells are notoriously resistant to transfection with exogenous mRNAs. Combining CD4 antibodies with lipid nanoparticles (LNPs) targets CD4+ cells, including T cells [155]. Moreover, ionizable lipid nanoparticle-mediated mRNA delivery for human CAR T cell engineering is also advancing [156]. In addition, B cells play important roles in shaping the initial expansion of CD4+ T cells and the memory of CD4+ T cells [157]. However, there are few relevant studies and in-depth studies are needed in the future.

## Conclusions and outlooks

Engineered NPs are ideal tools for the application of liver fibrosis therapy. However, only a few have been successfully translated into clinical practice, mainly due to the macrophage uptake of NPs. Disulfide bonds are commonly used as intermediate linkers in the fabrication of silicon networks, and disulfide-bonded organosilicon NPs with cage-like morphology target LSECs to avoid macrophage filtration and may affect the tolerance status of intrahepatic immune cells [158]. (1) Targeting specific cell types is expected to be a useful technique, suggesting the use of nanomedicines for diagnostic, therapeutic, and prognostic integration of personalized treatment of liver fibrosis is an important future drug development direction. LNPs accumulate in the LSECs to cause activation and neutrophil inflammation. Furthermore, the modification of N-acetyl-D-galactosamine (GalNAc)-LNPs with polyethylene glycol eliminates the toxicity associated with LNPs [159]. (2) Combination therapies with different mechanisms can be used for the development of engineered nanotherapeutics. The microenvironment of liver fibrosis is the result of multiple cellular interactions upon encountering the capillaries LSEC barrier, HA-NPs/SMV rapidly released SMV and exerted a fenestrae-repairing function, which allowed more CV-NPs/siCol1α1 to enter the space of Disse to degrade deposited collagen and finally to achieve higher accumulation in activated HSCs, promoting pathological barrier-normalization [32]. (3) The biosafety of the NPs should be investigated more. Although the biocompatibility of the NPs have been confirmed in *vitro* or in *vivo*. But when they need to be transferred to the clinical trials, such as the biodistribution after the injection, or the biodegradation pathway in different organs.

In addition to traditional drug therapies, nano vaccines allow precise modulation of the composition and structure of NPs, their physicochemical properties (size, shape, function, and surface charge) as well as the dose and route of administration of nano drugs, to improve antigen presentation and strong immunogenicity. Therefore, it has become an attractive alternative or complementary therapy in the field of cancer treatment [160] and has received lots of attention in the treatment of liver fibrosis. Polystyrene NPs, PLGA, CNTs, aluminum hydroxide NPs, SiO2 NPs, carbon black NPs, and TiO2 NPs, have been shown to stimulate NLRP3-related inflammasomes [161], and liposomes, polymers, and inorganic NPs, as well as self-assembled protein NPs and virus-like particles (VLPs), are being explored as antigen carriers [160]. Furthermore, achieving subcellular localization of drug loading is critical to maximizing the therapeutic potential of a drug [162]. Carbon dots (CDs) are new fluorescent nanomaterials with negligible photobleaching, among which graphene quantum dots (GQDs) can be used for super-selective cell nuclear imaging due to their superior biocompatibility and targeting ability [163]. In addition, quantum dots (QDs) are used for imaging and drug-targeted delivery, which allow rapid absorption in the small intestine after oral administration and is highly specific targeting to LSECs or hepatocytes [164]. Moreover, graphene oxide (GO) enables efficient liver regeneration via immunomodulation [43].

Meanwhile, the hepatic fibrosis microenvironment, composed of immune cells, MFs, LSECs, HSCs, and ECM with abundant growth/signaling factors, is a unique, complex, and highly dynamic region. These diverse cell types and ECM proteins are capable of coordinating liver remodeling, hematopoiesis, regulation of immune function, and tissue regeneration. Reprogramming the tumor microenvironment (TIME) and reversing immunosuppressive strategies are currently the most beneficial modalities for cancer therapy [165]. In addition, the hepatic fibrosis microenvironment and reversal of immunosuppression are the most promising strategies for liver fibrosis treatment. Moreover, the energy utilization of immune cells differs significantly in T cells exhibiting completely different metabolic patterns depending on the activation state [166]. For example, the metabolism of naive T cells is essentially static [167], exhibiting zero proliferation, therefore, only minimal nutrient intake. Minimal glycolytic rates and minimal biosynthesis are required to maintain reliance on OXPHOS to provide ATP [168]. As it becomes metabolically activated, there is an increased nutrient uptake, enhanced glycolysis rate, synthesis, and accumulation of proteins, lipids, and nucleotides, as well as the growth and proliferation of T cells to perform killing functions [168]. Memory T cells have a similar metabolic pattern to naive T cells, maintaining a basic nutrient intake, a lower glycolytic rate, and relying on OXPHOS for ATP [169]. In addition, activated NK cells [170], neutrophils [171], M1 macrophages [172], and DCs [173] rely mainly on glycolysis for energy supply. However, DCs used mainly oxidative phosphorylation for energy metabolism at resting state. In addition, aerobic glycolysis and pentose phosphate pathway are the main metabolic modes of neutrophils [174]. More interestingly, glycolysis and mitochondrial metabolism are enhanced following the activation of B lymphocytes induced by LPS stimulation or antigenic stimulation. However, glycolysis is the main metabolism in activated B lymphocytes [175]. However, Treg cells [176] and M2 macrophages [177] mainly rely on OXPHOS (FAO) from fatty acid oxidation for energy supply. It is suggested that novel immune cell metabolic reprogramming targeting biodegradability, specific selectivity, responsive drug release, and multimodal synergistic therapy of engineered nanomaterials have broad applications in the treatment of liver fibrosis.

Nanomedicine for fibrosis offers the opportunity to enhance the anti-fibrotic immune response, achieve specificity and local amplification of the immune response safely and effectively in fibrotic tissue, and improve the rate of patients` treatment for immunotherapy, as well as reduce related side effects. Several nanoparticle-based T-cell therapies remained unexploited. For example, more studies in recent years have identified new subpopulations of immune cells [178] and these subgroups are considered to be homogeneous groups, which may be a new target for immunotherapy, and the mechanisms of these therapies need to be further understood. The ability to manufacture "generic" or "off-the-shelf" NPs using antigen diagnostics will reduce the cost burden and expand the range of patients to be effectively treated with T-cell immunotherapy compared to cell-based therapies. In addition, multi-reactive nanomedicine for immunotherapy comprehensively modulates complex pathogenic processes. Therefore, NP-mediated T-cell immunotherapy will have sustained progress in the future.

## Data Availability

Not applicable.

## Abbreviations

APCs: Antigen-presenting cells
APRI: AST-to-platelet ratio index
ASO: Antisense oligonucleotides
AST: Aspartate aminotransferase
CAFs: Cancer-associated fibroblasts
CDs: Carbon dots
CB1R: Cannabinoid receptor-1
ChiBil: Chitosan-bilirubin micelle
CLD: Chronic liver disease
CLRs: C-type lectin receptors
CMC: Carboxymethyl chitosan
CNF: Cellulose nanofiber
ConA: Concanavalin A
COVID-19: Coronavirus pneumonia
ECM: Extracellular matrix
EO: Ethylene oxide
FAO: Fatty acids for mitochondrial β-oxidation
FDA: Food and Drug Administration
FIB-4: Fibrosis-4 index
FPL: Fulleropyrrolidine
GLN: Glutamine
GLUD1: Glutamate dehydrogenase
GO: Graphene oxide
GOT1: Aspartate aminotransferase
GQDs: Graphene quantum dots
HA: Hematoxylic acid
HCC: Hepatocellular carcinoma
IHVR: Intrahepatic vascular resistance
IL: Interleukin-10
KCs: Kupffer cells
LF: Liver Fibrosis test
LSECs: Liver sinusoidal endothelial cells
LNPs: Lipid nanoparticles
MFs: Myofibroblastic
mGluR5: Metabotropic glutamate receptor-5
MHC: Major histocompatibility complex
mLNP-siHMGB1: Mannose-modified HMGB1-siRNA
MR: Mannose receptors
MTC: Mannose-modified trimethyl chitosan-cysteine
M6P/IGFII: Mannose-6-phosphate/insulin-like growth factor II
NASH: Nonalcoholic steatohepatitis
NAS: NAFLD score
NKT cells: Natural killer T cells
NO: Nitric oxide
NPs: Nanoparticle
OXPHOS: Oxidative phosphorylation
PDK3: Pyruvate dehydrogenase kinase 3
PEI: Polyethyleneimine
PEG: Polyethylene glycol
PVA: Polyvinyl alcohol
PKM2: Pyruvate kinase M2
PLGA: Poly (lactic-co-glycolic acid)
PMs: Polymeric micelles
PO: Propylene oxide
pro-MMPs: Pro-matrix metalloproteinases
PPARγ: Peroxisome proliferator-activated receptor γ
PS: Phosphatidylserine
P5C: Pyrroline-5-carboxylic acid
QDs: Quantum dots
RAP: Allowing RcP carriers
RBP4: Particularly retinol-binding protein 4
RcP: Further binds to nucleotides
RES: Reticuloendothelial system
RNAi: RNA interference
SLNs: Solid lipid NPs
SPION: Suoxide pellets
SR: Scavenger receptors
stab2: Scavenger receptor stabilizer-2
TIME: Tumor microenvironment
TLR: Toll-like receptor
Tregs: Regulatory T cells
USSN: Ultrasmall silica NPs
VLPs: Virus-like particles
VA: Vitamin A
2-AG: 2-Arachidonic acid glycerol
α-KG: α-Ketoglutarate
