# Correction: Remodeling the hepatic fibrotic microenvironment with emerging nanotherapeutics: a comprehensive review

**DOI:** 10.1186/s12951-023-01914-2

**Published:** 2023-05-12

**Authors:** Xingtao Zhao, Felix Kwame Amevor, Xinyan Xue, Cheng Wang, Zhifu Cui, Shu Dai, Cheng Peng, Yunxia Li

**Affiliations:** 1 State Key Laboratory of Southwestern Chinese Medicine Resources, Key Laboratory of Standardization for Chinese Herbal Medicine, Ministry of Education, Chengdu university of Traditional Chinese Medicine, Chengdu, China; 2 School of Pharmacy, Chengdu University of Traditional Chinese Medicine, Chengdu, 611137 China; 3 Farm Animal Genetic Resources Exploration and Innovation Key Laboratory of Sichuan Province, Sichuan Agricultural University, Chengdu, 611130 China; 4 College of Animal Science and Technology, Southwest University, Chongqing, 400715 China

**Correction: Zhao** ***et al. Journal of Nanobiotechnology (2023) 21:121***

10.1186/s12951-023-01876-5

Following publication of the original article [1], the authors identified an error in the affiliations. The corrected affiliation 1 is given below and affiliation 5 was removed.

State Key Laboratory of Southwestern Chinese Medicine Resources, Key Laboratory of Standardization for Chinese Herbal Medicine, Ministry of Education, Chengdu university of Traditional Chinese Medicine, Chengdu, China.

The original article has been corrected.
